# Gastric Tissue Biomonitoring Identifies Tissue-Specific Metal Profiles Associated with *Helicobacter pylori* Infection

**DOI:** 10.3390/toxics14090808

**Published:** 2026-09-11

**Authors:** Tolga Aydin, Vugar Ali Turksoy

**Affiliations:** 1 Department of Internal Medicine, Faculty of Medicine, Yozgat Bozok University, 66100 Yozgat, Türkiye; tolga.aydin@yobu.edu.tr; 2 Department of Public Health, Faculty of Medicine, Yozgat Bozok University, 66100 Yozgat, Türkiye

**Keywords:** *Helicobacter pylori*, toxic metals, aluminum, arsenic, cobalt, ICP-MS, gastric tissue, biomonitoring, one health perspective

## Abstract

**Background**: *Helicobacter pylori* infection is a common chronic bacterial infection and an established cause of gastritis, peptic ulcer disease, and gastric cancer. Environmental and host metal status may interact with gastric inflammatory and microbial processes, and infection-related changes in gastric acidity, mucosal permeability, and metal handling may in turn alter local tissue concentrations. This study compared systemic and gastric tissue concentrations of seven elements in adults with histopathologically confirmed *H. pylori* infection and uninfected controls. **Methods**: This single-center case–control study included 110 adults undergoing upper gastrointestinal endoscopy (55 *H. pylori*-positive and 55 *H. pylori*-negative). *H. pylori* status was established by hematoxylin–eosin and modified Giemsa staining. Whole blood collected in purple-top K_2_EDTA tubes was analyzed for As, Pb, Cd, and Sn; serum obtained from yellow-top serum-separator tubes containing clot activator and separator gel was analyzed for Al, Co, and Ni. A separate fresh antral biopsy, not the formalin-fixed histology specimen, was used for ICP-MS; tissue concentrations were normalized to recorded dry weight. Between-group metal comparisons were corrected as one family of 14 tests using Benjamini–Hochberg FDR. Prespecified age- and sex-adjusted models and an exploratory multivariable model were supplemented by ROC, PCA, and internally cross-validated PLS-DA analyses. **Results**: The *H. pylori*-positive group was older than the negative group (55.65 ± 15.01 vs. 52.42 ± 12.53 years). Blood aluminum was nominally higher in the positive group (*p* = 0.032; q = 0.112). In gastric tissue, aluminum was higher [0.23 (0.13–13.40) vs. 0.13 (0.10–0.23) μg/g dry weight; *p* < 0.001; q = 0.002], whereas arsenic was lower [1.35 (0.54–2.02) vs. 1.92 (1.47–2.43) μg/g dry weight; *p* < 0.001; q = 0.003] in *H. pylori*-positive participants. Tissue cobalt and blood/tissue nickel differences did not remain significant after FDR correction. In prespecified age- and sex-adjusted models, tissue arsenic showed an inverse association (OR = 0.431, 95% CI 0.263–0.705; *p* = 0.0008), whereas tissue aluminum showed a positive association (OR = 4.957, 95% CI 1.629–15.085; *p* = 0.0048). In the exploratory joint model, only tissue aluminum remained significant (adjusted OR = 4.016, 95% CI 1.119–14.417; *p* = 0.033). The joint model had an apparent within-cohort AUC of 0.774; repeated five-fold cross-validated PLS-DA yielded an AUC of 0.707. **Conclusions**: Histopathologically confirmed *H. pylori* status was associated with compartment-specific metal concentration patterns, particularly higher tissue aluminum and lower tissue arsenic. Because the study was cross-sectional and measured total concentrations, these findings do not establish environmental exposure, temporal accumulation, toxicity, or a causal direction. The regression and multivariate findings are exploratory and require validation in independent cohorts with standardized tissue sampling, arsenic speciation, exposure assessment, and quantitative gastric pathology.

## 1. Introduction

*Helicobacter pylori* (*H. pylori*) infection remains one of the most prevalent chronic bacterial infections worldwide and represents a major determinant of gastric mucosal inflammation, peptic ulcer disease, gastric atrophy, intestinal metaplasia, mucosa-associated lymphoid tissue lymphoma, and gastric adenocarcinoma [1,2,3,4]. Because of its causal role in non-cardia gastric cancer, *H. pylori* has been classified by the International Agency for Research on Cancer as a Group 1 human carcinogen [5]. Nevertheless, only a subset of infected individuals develops severe gastric pathology, suggesting that bacterial virulence factors alone are insufficient to explain disease progression. Host immunity, nutritional status, environmental exposures, socioeconomic factors, gastric microbiota composition, and toxic metal burden may all contribute to the biological heterogeneity of *H. pylori*-associated disease [6,7,8,9].

The exposome concept provides a framework for considering environmental exposures across the life course [10,11,12]. Metals and metalloids are widespread in food, water, air, tobacco smoke, and occupational settings, but their biological relevance depends on dose, chemical species, exposure timing, and the matrix measured [13,14,15,16,17,18,19,20,21]. These factors are particularly important in gastric tissue, where a measured total concentration may reflect recent luminal contact, epithelial uptake, systemic delivery, or altered local handling. Metal homeostasis is also central to *H. pylori* biology. Nickel is required for maturation of urease, which supports acid acclimation, and for [NiFe]-hydrogenase, which permits use of molecular hydrogen as an energy source. *H. pylori* regulates nickel import, trafficking, storage, and toxicity through NixA, NiuBDE, accessory proteins, histidine-rich proteins, and the NikR regulon [22,23,24,25,26].

A growing body of evidence suggests that some metals can modify innate and adaptive immune functions [18,27,28,29,30]. Epidemiological studies have reported associations between blood lead or cadmium and chronic infections, including *H. pylori*, although residual confounding and cross-sectional design limit causal interpretation [29,31]. Conversely, *H. pylori*-associated gastritis can change gastric acidity, epithelial permeability, transporter activity, and micronutrient absorption [32,33,34]. The relation is therefore potentially bidirectional: antecedent metal exposure or host metal status could influence the gastric microenvironment, while infection could alter the distribution or retention of elements. Prior element studies have been inconsistent; for example, Hu et al. found higher serum cobalt among infected participants but no consistent differences for most other measured elements [35].

Environmental toxicants can alter microbial communities and host inflammatory signaling, and *H. pylori* itself reshapes the gastric ecosystem [7,8,9,36,37,38,39,40,41,42,43]. These observations provide biological context but were not evaluated in the present study. Likewise, although several metals and *H. pylori* are independently linked to carcinogenic pathways [44,45,46], the current data do not test microbiome alteration, oxidative stress, epithelial injury, or gastric carcinogenesis. These mechanisms are therefore considered only as hypotheses for future work.

A One Health and exposome perspective may help integrate environmental, dietary, microbial, and host determinants of gastric disease [10,11,12,47,48]. ICP-MS supports sensitive multi-element measurement in biological matrices [49,50,51], but tissue biomonitoring requires careful interpretation because concentration does not by itself identify exposure source, duration, biological availability, or toxicity. Important evidence gaps include the limited use of histopathologically confirmed active infection, the scarcity of paired blood-tissue measurements, and the lack of standardized reporting for gastric biopsy collection and weight normalization.

Therefore, this study aimed to compare concentrations of seven elements in blood-derived matrices and fresh gastric tissue between adults with and without histopathologically confirmed *H. pylori* infection. Secondary aims were to evaluate age- and sex-adjusted associations and explore whether multi-element profiles discriminated infection status within the study cohort. We hypothesized that measured metal profiles would differ between groups; no temporal or causal direction was prespecified.

## 2. Materials and Methods

### 2.1. Study Design and Population

**Figure 1:**
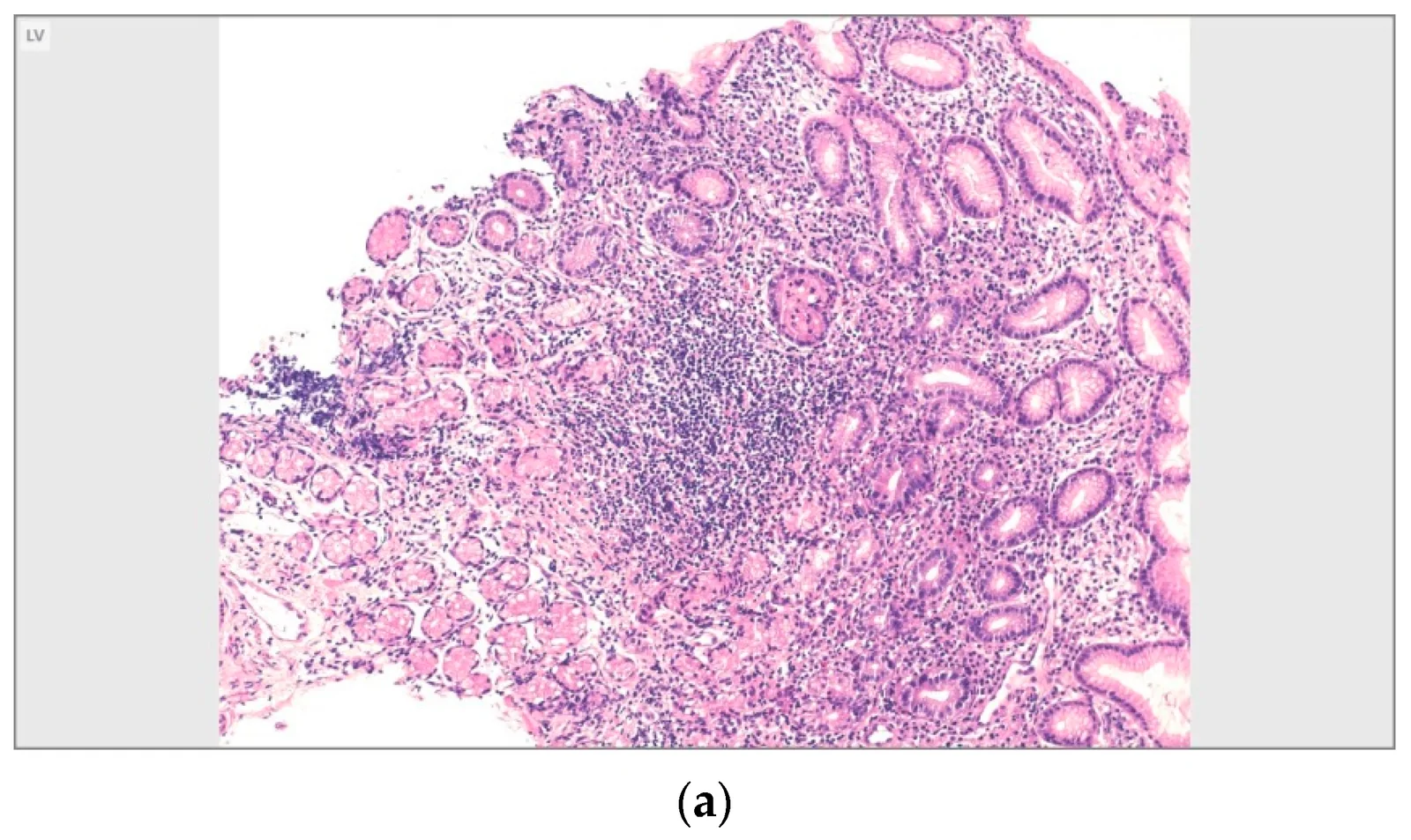

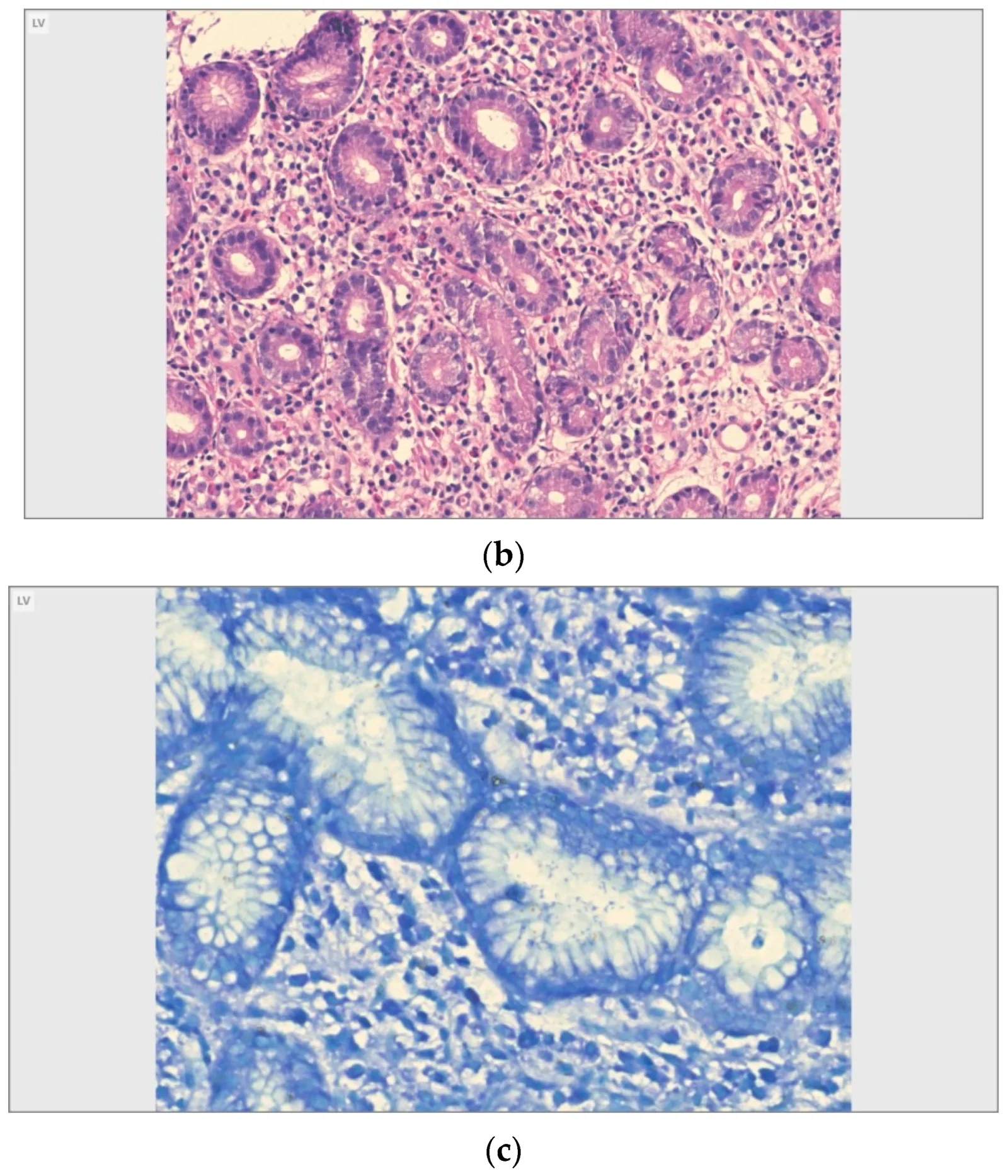
Representative histopathological images used to confirm *H. pylori* infection. (**a**) A prominent lymphoid aggregate with lymphocyte- and plasma-cell-predominant chronic inflammatory infiltration between antral glands (H&E, ×100). (**b**) Antral mucosa with neutrophilic infiltration in the lamina propria, indicating active inflammation (H&E, ×200). (**c**) Curved and spiral bacillary forms within antral glands (modified Giemsa, ×400). These images illustrate qualitative diagnostic confirmation; histopathological severity was not quantitatively scored.

This single-center observational case–control study was conducted at the Department of Internal Medicine and Endoscopy Unit of Yozgat Bozok University Faculty of Medicine, Türkiye. The primary objective was to investigate whether systemic and local gastric heavy metal concentrations differed between patients with and without *H. pylori* infection. The study included adult patients who underwent diagnostic upper gastrointestinal endoscopy because of dyspeptic symptoms. Patients were consecutively recruited between February 2025 and October 2025. Following histopathological evaluation (Figure 1), participants were allocated into two groups according to *H. pylori* status. The final study population consisted of 110 individuals, including 55 patients with histopathologically confirmed *H. pylori* infection and 55 *H. pylori*-negative controls. Patients younger than 18 years of age, those with previous gastric surgery, gastric malignancy, inflammatory bowel disease, chronic liver failure, end-stage renal disease, autoimmune diseases, active gastrointestinal bleeding, pregnancy, previous heavy metal chelation therapy, occupational heavy metal intoxication, or incomplete clinical data were excluded.

### 2.2. Ethical Approval

The study protocol was approved by the Yozgat Bozok University Non-Interventional Clinical Research Ethics Committee (Decision No. 2025-GOKAEK-253_2025.02.05_346, Approval Date: 5 February 2025). The study was conducted according to the ethical principles of the Declaration of Helsinki and Good Clinical Practice guidelines. All patients provided written informed consent before upper gastrointestinal endoscopy. Biological materials used in the present study were obtained during routine diagnostic procedures and preoperative assessments; no additional invasive intervention was performed for research purposes.

### 2.3. Clinical Evaluation and Histopathological Diagnosis

Demographic characteristics, including age, sex, body mass index (BMI), smoking status, alcohol consumption, comorbidities, medication history, routine laboratory findings, and endoscopic reports, were obtained from the hospital electronic medical record system. All patients underwent upper gastrointestinal endoscopy performed by experts using standard clinical procedures, following a strict minimum preoperative fasting period of 10 h to minimize confounding from recent dietary intake. During endoscopy, gastric mucosal biopsy specimens were routinely obtained from both the antrum and corpus in accordance with current clinical practice. Biopsy specimens were immediately fixed in 10% neutral buffered formalin, processed routinely, embedded in paraffin, sectioned at a thickness of 4 μm, and stained with hematoxylin–eosin (H&E) and modified Giemsa stain for histopathological examination. Histopathological assessments were independently performed by experienced pathologists who were blinded to all clinical characteristics and heavy metal measurements. Patients demonstrating microscopic evidence of *H. pylori* colonization were classified as *H. pylori*-positive, whereas those without histopathological evidence of bacterial colonization were classified as *H. pylori*-negative. Histopathological diagnosis was selected as the reference method because it directly demonstrates active bacterial colonization of the gastric mucosa and provides greater diagnostic specificity than serological methods.

### 2.4. Blood and Gastric Tissue Sampling

Peripheral venous blood samples were collected immediately before endoscopy during routine clinical phlebotomy. For As, Pb, Cd, and Sn, whole blood was collected in purple-top K_2_EDTA tubes and was not centrifuged before digestion. For Al, Co, and Ni, blood was collected in yellow-top serum-separator tubes containing clot activator and separator gel; after clot formation, samples were centrifuged at 3000 rpm for 10 min and serum was transferred into acid-washed polypropylene tubes. A 1.0 mL aliquot of the relevant matrix was used for digestion. The terms whole blood and serum are used explicitly throughout the manuscript rather than the nonspecific term blood fraction.

Biopsies required for routine histopathology were fixed in 10% neutral-buffered formalin and were not used for elemental analysis. A separate fresh antral mucosal biopsy was collected for ICP-MS. The analytical biopsy was gently rinsed with trace-metal-free Triton X-100 solution followed by ultrapure water to reduce mucus, residual gastric contents, and loosely adherent surface material, then transferred to a sterile trace-metal-free polypropylene cryovial. The analytical specimen was not pooled with corpus tissue, and no formalin-fixed or paraffin-embedded material was analyzed by ICP-MS.

Whole blood, serum, and fresh antral biopsy specimens were transported under continuously monitored temperature conditions to the Yozgat Bozok University Science and Technology Application and Research Center (BILTEM), Yozgat, Türkiye, and stored at −80 °C until preparation. Powder-free nitrile gloves, acid-washed polypropylene materials, ultrapure reagents, procedural blanks, and standardized clean-handling procedures were used throughout collection, transport, storage, and analysis.

### 2.5. Determination of Heavy Metal Concentrations by ICP-MS

Concentrations of seven elements—aluminum (Al), arsenic (As), cadmium (Cd), cobalt (Co), nickel (Ni), lead (Pb), and tin (Sn)—were quantified using inductively coupled plasma-mass spectrometry (Thermo Scientific iCAP Q, Thermo Fisher Scientific, Waltham, MA, USA). For liquid matrices, 1.0 mL whole blood or serum was transferred to an acid-washed digestion vessel. For tissue, the entire fresh analytical biopsy was dried to constant weight and weighed; biopsy yield varied, so no fixed nominal tissue mass was imposed. The recorded dry mass was used as the denominator for μg/g dry-weight concentrations. Each specimen was combined with 5 mL ultrapure nitric acid (65%, Suprapur, Merck, Darmstadt, Germany), 2 mL hydrogen peroxide (30%), and 3 mL ultrapure water, pre-digested for 24 h at room temperature, microwave digested, and diluted to 10 mL. Instrument settings were RF power 1550 W, plasma gas flow 0.87 L/min, nebulizer gas flow 0.96 L/min, nebulizer pressure 3.02 bar, spray-chamber temperature 3.7 °C, and dwell time 0.01 ms. The sample introduction system was washed sequentially with ultrapure water (30 s), 2% nitric acid (45 s), and ultrapure water (45 s). Eleven-point external calibration (0.1–250 μg/L) achieved R^2^ > 0.999 for every reported element [52].

### 2.6. Quality Assurance and Quality Control

Comprehensive QA/QC procedures were applied. Samples and calibration standards were measured in quintuplicate and the mean was used; analytical RSDs were <5%. Seronorm Trace Elements Whole Blood Level-1 certified reference material (Sero AS, Billingstad, Norway) was used to verify accuracy, and hafnium (100 μg/L) served as the internal standard. Reagent blanks, procedural blanks, duplicate samples, calibration-verification standards, and intra-/inter-day precision checks were included in each analytical batch. Tissue wet and dry weights were recorded, but reported concentrations were calculated using the measured dry weight. Histology specimens and analytical biopsies were handled as separate sample streams, and formalin-fixed material was never introduced into the elemental-analysis workflow.

### 2.7. Statistical Analysis

All conventional statistical analyses were performed in IBM SPSS Statistics version 26.0 (IBM Corp., Armonk, NY, USA). Distributional assumptions were assessed using Shapiro–Wilk tests, histograms, and Q-Q plots. Normally distributed variables are presented as mean ± SD and compared with independent-samples t tests; non-normal variables are presented as median (IQR) and compared with Mann–Whitney U tests (Table 1). Categorical variables were compared with Pearson chi-square or Fisher exact tests. For the primary between-group metal analysis, Benjamini–Hochberg FDR correction was applied to one explicitly defined family of 14 comparisons (seven elements in their specified blood-derived matrix plus the same seven elements in gastric tissue) (Table 2). The seven matched blood-tissue Spearman correlations constituted a separate FDR family. Both unadjusted *p* values and adjusted q values are reported. *H. pylori*-positive status was coded 1 and negative status 0. Metal concentrations were transformed as ln(x + 1) and standardized; ORs therefore represent a 1-SD increase. Prespecified core logistic models adjusted each metal for age and sex; these age- and sex-adjusted estimates are reported in a dedicated column of Table 3, separately from the univariate estimates and from the exploratory joint model. The exploratory joint model simultaneously included age, sex, and metals with univariate logistic *p* < 0.10 (blood Al, blood Ni, tissue As, and tissue Al); no automated stepwise deletion was used. ORs, 95% CIs, *p* values, calibration, collinearity, and events per variable are reported in Table 3. ROC results are described as apparent within-cohort discrimination rather than diagnostic accuracy. All tests were two-sided; *p* < 0.05 defined nominal statistical significance.

Multivariate analyses were performed in Python 3 using scikit-learn. Participant-level concentrations were transformed as ln(x + 1), centered, and scaled to unit variance; complete cases were analyzed without imputation. PCA was unsupervised, whereas PLS-DA used histopathological *H. pylori* status as the binary class label. Two components were prespecified for visualization and for the internally validated PLS-DA model. Performance was estimated from out-of-fold probabilities generated by stratified repeated five-fold cross-validation. The cross-validated AUC and 95% CI were supplemented by 1000 label permutations in which the complete cross-validation procedure was repeated. These analyses assess exploratory within-cohort discrimination only and do not establish external predictive performance.

## 3. Results

A total of 110 participants were analyzed: 55 with histopathologically confirmed *H. pylori* infection and 55 *H. pylori*-negative controls. The positive group was older than the negative group (55.65 ± 15.01 vs. 52.42 ± 12.53 years). Women represented 60.0% (33/55) of the positive group and 54.5% (30/55) of the negative group (*p* = 0.531). Thus, sex distribution was similar, whereas age was not comparable and was included in adjusted models. Routine biochemical measurements are summarized in Table 1. No variable met the prespecified *p* < 0.05 threshold; calcium and TSH each had *p* = 0.050 and are described as borderline rather than significant.

**Table 1:** Comparison of biochemical parameters between *H. pylori*-positive and *H. pylori*-negative groups.

Parameters	*H. pylori* (+) (*n* = 55)	*H. pylori* (−) (*n* = 55)	*p*
HbA1c (%)	5.73 (5.51–5.99)	5.73 (5.33–6.11)	0.957
Glucose (mg/dL)	92.20 (87.40–107.60)	95.00 (89.20–110.30)	0.435
BUN (mg/dL)	13.40 (10.80–16.95)	13.30 (10.00–16.60)	0.727
Urea (mg/dL)	28.29 (23.01–35.42)	26.70 (20.88–33.70)	0.243
Creatinine (mg/dL)	0.77 (0.68–0.94)	0.74 (0.68–0.90)	0.851
Sodium (mmol/L)	139.00 (138.00–142.00)	140.00 (138.50–141.00)	0.873
Potassium (mmol/L)	4.35 (4.08–4.56)	4.32 (4.19–4.61)	0.437
ALT (U/L)	16.00 (12.50–22.40)	18.70 (15.00–24.65)	0.129
AST (U/L)	17.50 (14.85–19.00)	17.90 (15.60–20.80)	0.300
ALP (U/L)	80.00 (60.50–106.50)	81.50 (66.25–97.50)	0.835
Amylase (U/L)	69.22 ± 22.66	65.88 ± 21.51	0.486
GGT (U/L)	18.00 (12.75–27.00)	23.00 (15.00–35.00)	0.107
LDH (U/L)	189.50 (173.75–229.00)	181.50 (161.75–211.00)	0.263
Lipase (U/L)	30.55 (23.25–37.17)	28.50 (23.25–37.85)	0.916
Total protein (g/L)	70.70 (67.55–75.25)	72.40 (68.50–74.60)	0.451
Albumin (g/L)	44.95 (42.00–46.25)	44.20 (41.78–47.22)	0.953
Calcium (mg/dL)	9.34 (9.02–9.60)	9.58 (9.20–9.76)	0.050 *
Magnesium (mg/dL)	2.00 (1.90–2.09)	1.96 (1.87–2.04)	0.376
Phosphorus (mg/dL)	3.66 (3.01–3.94)	3.40 (3.17–3.67)	0.328
Direct bilirubin (mg/dL)	0.17 (0.14–0.22)	0.18 (0.14–0.22)	0.909
Total bilirubin (mg/dL)	0.47 (0.37–0.62)	0.49 (0.39–0.64)	0.619
Total cholesterol (mg/dL)	185.71 ± 49.42	195.83 ± 48.13	0.288
Triglycerides (mg/dL)	132.75 (95.00–182.57)	143.00 (94.60–214.80)	0.542
HDL cholesterol (mg/dL)	44.75 (36.90–53.65)	45.00 (40.53–55.80)	0.467
LDL cholesterol (mg/dL)	110.47 ± 35.85	115.31 ± 37.69	0.498
Serum iron (µg/dL)	72.68 (53.59–101.22)	67.97 (41.01–99.03)	0.359
TIBC (µg/dL)	283.46 ± 73.01	297.42 ± 75.21	0.328
Uric acid (mg/dL)	4.65 (3.85–5.60)	4.60 (3.74–5.50)	0.904
C-reactive protein (mg/L)	2.05 (0.94–4.93)	2.68 (1.15–4.45)	0.714
TSH (mIU/L)	1.33 (1.03–2.41)	1.73 (1.40–2.23)	0.050 *
Free T3	0.31 (0.29–0.38)	0.32 (0.29–1.36)	0.986
Free T4	1.23 (1.15–1.38)	1.21 (1.14–1.33)	0.324
Anti-TPO (IU/mL)	8.44 (2.70–50.05)	10.60 (10.34–10.78)	0.662
Anti-Tg (IU/mL)	10.25 (0.68–119.96)	7.38 (3.79–10.96)	0.800
Ferritin (ng/mL)	35.65 (22.30–95.56)	36.89 (18.73–68.70)	0.487
Vitamin B12 (pg/mL)	319.70 (243.75–410.60)	363.00 (289.10–447.00)	0.100
Vitamin D (ng/mL)	16.81 ± 9.53	18.39 ± 7.74	0.436

Two-sided *p* values are reported; *p* < 0.05 was considered statistically significant. Data are presented as median (IQR) or mean ± SD. * Borderline value (*p* = 0.050) that did not reach the prespecified significance threshold.

**Figure 2:**
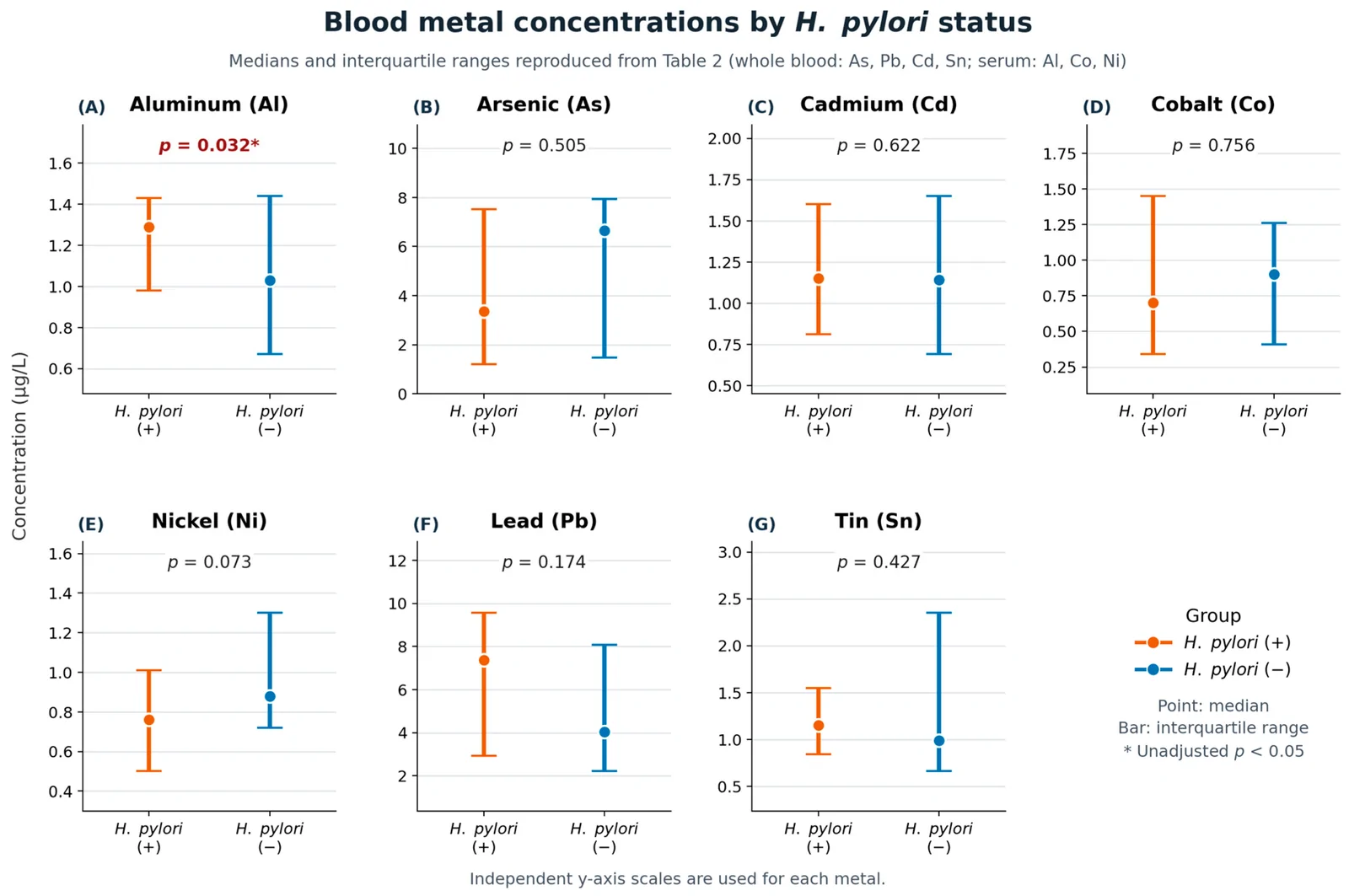
Concentrations of the seven elements in their specified blood-derived matrices by *H. pylori* status. Points show medians and bars show IQRs; displayed *p* values are unadjusted. * Unadjusted *p* < 0.05. No blood-derived comparison remained significant after FDR correction across the 14 primary comparisons.

**Figure 3:**
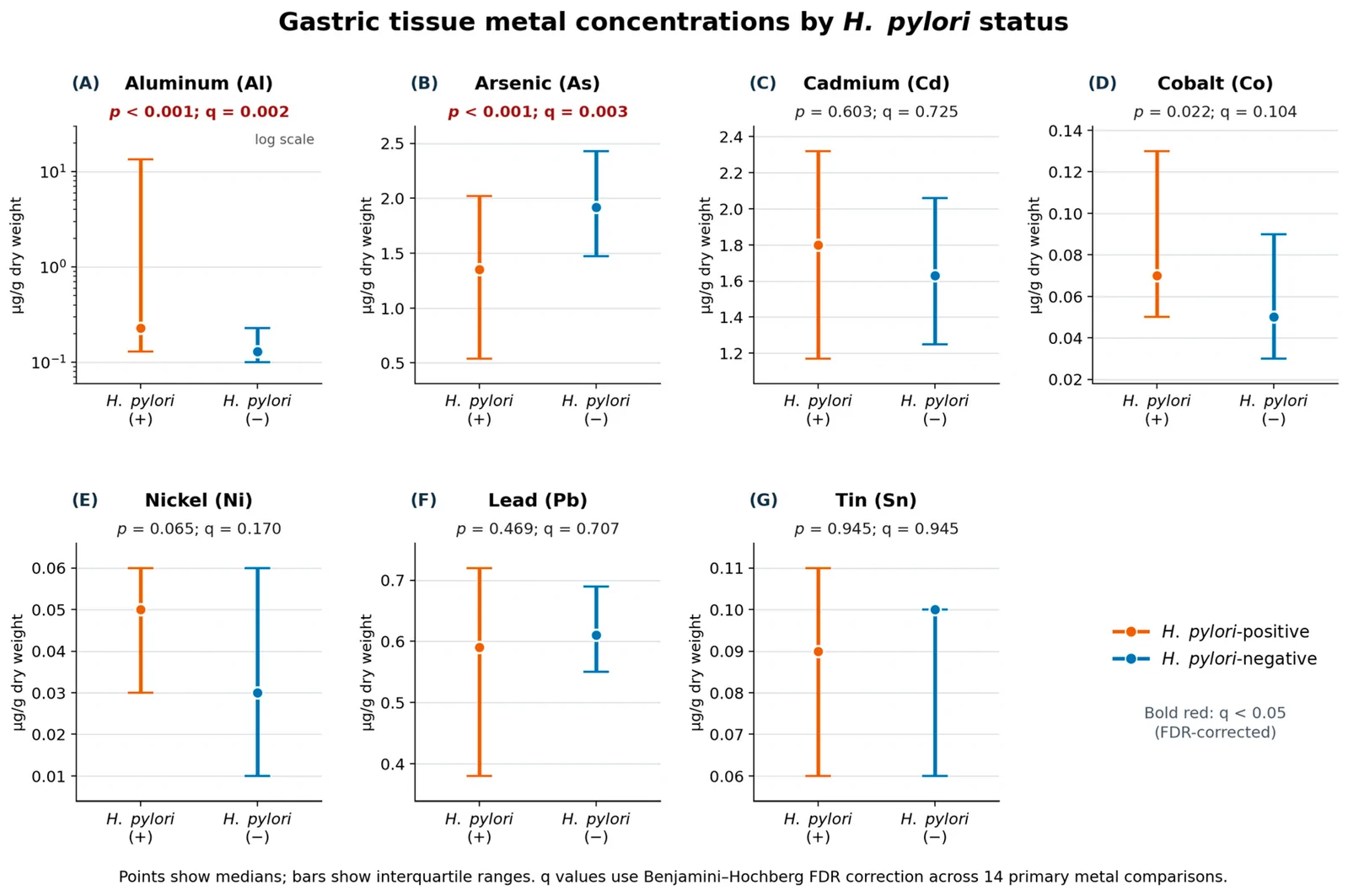
Gastric tissue concentrations of seven elements by *H. pylori* status. Points show medians and bars show IQRs; the aluminum panel uses a logarithmic y-axis because of marked right-skew. Unadjusted *p* values and Benjamini–Hochberg q values are shown. Values shown in bold red indicate q < 0.05. Tissue aluminum and tissue arsenic remained significant after correction across the 14 primary comparisons.

**Figure 4:**
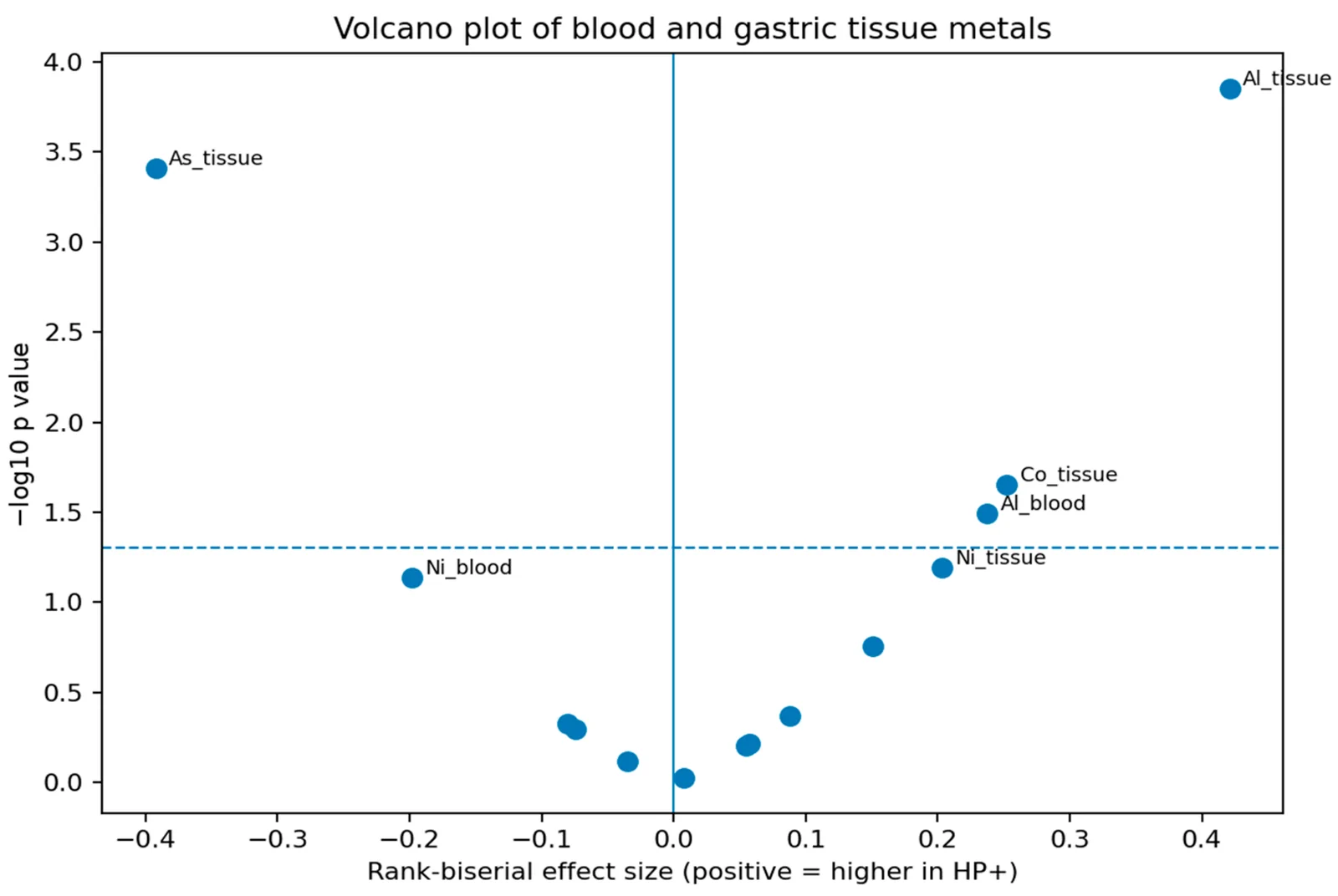
Volcano plot illustrating statistical significance and magnitude of differences in blood and gastric tissue metals. The x-axis shows the rank-biserial effect size (positive values indicate higher concentrations in the *H. pylori*-positive group, HP+), and the y-axis shows the −log10-transformed unadjusted *p* value. The horizontal dashed line indicates the nominal significance threshold (*p* = 0.05), and the vertical solid line indicates no between-group difference (effect size = 0).

**Figure 5:**
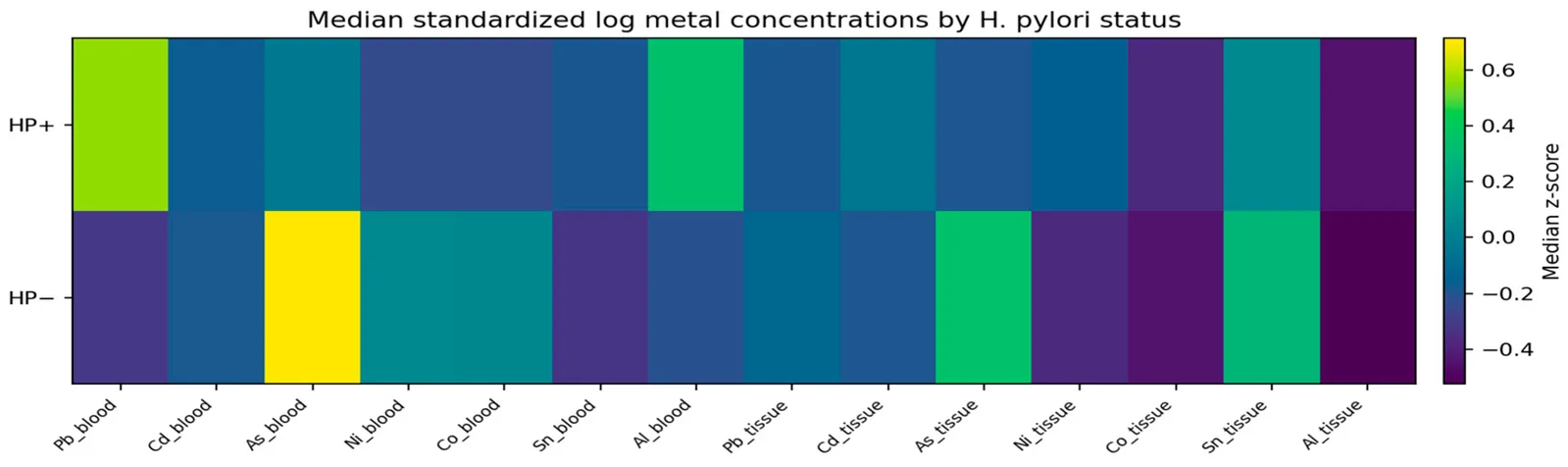
Heatmap of standardized median metal concentrations in blood-derived matrices and gastric tissue by *H. pylori* status.

Table 2 presents the verified medians, IQRs, unadjusted *p* values, and FDR-adjusted q values. Blood aluminum was nominally higher in *H. pylori*-positive participants [1.29 (0.98–1.43) vs. 1.03 (0.67–1.44) μg/L; *p* = 0.032], but it did not remain significant after correction across the 14 primary comparisons (q = 0.112). Blood nickel was numerically lower [0.76 (0.50–1.01) vs. 0.88 (0.72–1.30) μg/L; *p* = 0.073; q = 0.170]. In tissue, aluminum was higher [0.23 (0.13–13.40) vs. 0.13 (0.10–0.23) μg/g dry weight; *p* = 0.00014; q = 0.00196], whereas arsenic was lower [1.35 (0.54–2.02) vs. 1.92 (1.47–2.43) μg/g dry weight; U = 919.0; *p* = 0.00039; q = 0.00275] in the positive group. Tissue cobalt was nominally higher (*p* = 0.022; q = 0.104), and tissue nickel showed a borderline difference (*p* = 0.065; q = 0.170); neither survived FDR correction. No other comparison was significant after correction (Figure 2, Figure 3, Figure 4 and Figure 5).

**Table 2:** Comparison of the seven measured elements in blood-derived matrices and fresh gastric tissue according to *H. pylori* status.

Matrix	Element	*H. pylori* (+) (*n* = 55) Median (IQR)	*H. pylori* (−) (*n* = 55) Median (IQR)	*p*	FDR q
Whole blood/serum (μg/L)	Lead (Pb)	7.37 (2.92–9.56)	4.03 (2.21–8.08)	0.174	0.348
Whole blood/serum (μg/L)	Cadmium (Cd)	1.15 (0.81–1.60)	1.14 (0.69–1.65)	0.622	0.725
Whole blood/serum (μg/L)	Arsenic (As)	3.35 (1.21–7.51)	6.65 (1.47–7.93)	0.505	0.707
Whole blood/serum (μg/L)	Nickel (Ni)	0.76 (0.50–1.01)	0.88 (0.72–1.30)	0.073	0.170
Whole blood/serum (μg/L)	Cobalt (Co)	0.70 (0.34–1.45)	0.90 (0.41–1.26)	0.756	0.814
Whole blood/serum (μg/L)	Tin (Sn)	1.15 (0.84–1.55)	0.99 (0.66–2.35)	0.427	0.707
Whole blood/serum (μg/L)	Aluminum (Al)	1.29 (0.98–1.43)	1.03 (0.67–1.44)	0.032	0.112
Fresh gastric tissue (μg/g dry weight)	Lead (Pb)	0.59 (0.38–0.72)	0.61 (0.55–0.69)	0.469	0.707
Fresh gastric tissue (μg/g dry weight)	Cadmium (Cd)	1.80 (1.17–2.32)	1.63 (1.25–2.06)	0.603	0.725
Fresh gastric tissue (μg/g dry weight)	Arsenic (As)	1.35 (0.54–2.02)	1.92 (1.47–2.43)	<0.001	0.003 *
Fresh gastric tissue (μg/g dry weight)	Nickel (Ni)	0.05 (0.03–0.06)	0.03 (0.01–0.06)	0.065	0.170
Fresh gastric tissue (μg/g dry weight)	Cobalt (Co)	0.07 (0.05–0.13)	0.05 (0.03–0.09)	0.022	0.104
Fresh gastric tissue (μg/g dry weight)	Tin (Sn)	0.09 (0.06–0.11)	0.10 (0.06–0.10)	0.945	0.945
Fresh gastric tissue (μg/g dry weight)	Aluminum (Al)	0.23 (0.13–13.40)	0.13 (0.10–0.23)	<0.001	0.002 *

Unadjusted *p* values are from two-sided Mann–Whitney U tests. FDR q values were calculated by the Benjamini–Hochberg procedure across one family of 14 primary comparisons (seven blood-derived and seven tissue comparisons). q < 0.05 was considered significant after correction. Whole blood was used for As, Pb, Cd, and Sn; serum was used for Al, Co, and Ni. Bold values marked with * indicate q < 0.05 (significant after FDR correction).

In the prespecified age- and sex-adjusted models, each 1-SD increase in ln(x + 1)-transformed tissue arsenic was associated with lower odds of *H. pylori* positivity (OR = 0.431, 95% CI 0.263–0.705; *p* = 0.0008), whereas tissue aluminum was associated with higher odds (OR = 4.957, 95% CI 1.629–15.085; *p* = 0.0048). In the same prespecified framework, blood aluminum (OR = 1.459, 95% CI 0.962–2.215; *p* = 0.076) and blood nickel (OR = 0.686, 95% CI 0.454–1.036; *p* = 0.073) were no longer nominally significant once age and sex were taken into account. The outcome was coded positive = 1 and negative = 0. In the exploratory joint model that simultaneously included age, sex, blood aluminum, blood nickel, tissue arsenic, and tissue aluminum, only tissue aluminum remained significant (adjusted OR = 4.016, 95% CI 1.119–14.417; *p* = 0.033). Table 3 reports, for every candidate variable, the univariate estimate, the prespecified age- and sex-adjusted estimate, and the exploratory joint-model estimate, each with its 95% CI and *p* value.

**Table 3:** Univariate and exploratory multivariable logistic regression for *H. pylori* positivity.

Candidate Variable	Univariate OR (95% CI)	*p*	Age- and Sex-Adjusted OR (95% CI)	*p*	Final Adjusted OR (95% CI)	*p*
Age	1.039 (1.009–1.069)	0.009	1.030 (0.997–1.065)	0.182	1.021 (0.987–1.055)	0.227
Male sex	0.741 (0.347–1.585)	0.440	0.738 (0.297–1.641)	0.448	0.716 (0.288–1.781)	0.473
Blood aluminum	1.604 (1.066–2.415)	0.023	1.459 (0.962–2.215)	0.076	1.377 (0.895–2.119)	0.146
Blood nickel	0.659 (0.441–0.984)	0.041	0.686 (0.454–1.036)	0.073	0.771 (0.485–1.224)	0.270
Tissue arsenic	0.443 (0.277–0.709)	<0.001	0.431 (0.263–0.705)	0.0008	0.724 (0.409–1.281)	0.267
Tissue aluminum	5.321 (1.774–15.959)	0.003	4.957 (1.629–15.085)	0.0048	4.016 (1.119–14.417)	0.033

OR, odds ratio; CI, confidence interval. *H. pylori*-positive was coded 1 and negative 0. Metal concentrations were transformed as ln(x + 1) and standardized; ORs are per 1-SD increase. The age- and sex-adjusted column reports the prespecified single-metal models, in which each metal was entered together with age and sex only; age and sex are the adjustment covariates of these models and therefore have no separate entry in that column. The exploratory final model simultaneously included age, sex, blood aluminum, blood nickel, tissue arsenic, and tissue aluminum. Age and sex were prespecified covariates; metals entered the candidate set when univariate *p* < 0.10. No automated stepwise deletion was used. Events per variable = 9.2; maximum VIF = 2.14; Hosmer–Lemeshow *p* = 0.815; Nagelkerke R^2^ = 0.374; apparent AUC = 0.774.

Receiver operating characteristic analysis was used only to describe exploratory within-cohort discrimination. The joint exploratory logistic model had an apparent AUC of 0.774 (95% CI 0.682–0.854), sensitivity of 69.1%, and specificity of 78.2% at the Youden cut-off of 0.423. Individual apparent AUCs were 0.711 for tissue aluminum, 0.696 for tissue arsenic (oriented so that lower arsenic predicted positivity), 0.626 for tissue cobalt, and 0.619 for blood aluminum. Because development and evaluation used the same cohort, these estimates may be optimistic and are not interpreted as diagnostic utility or external predictive accuracy.

**Figure 6:**
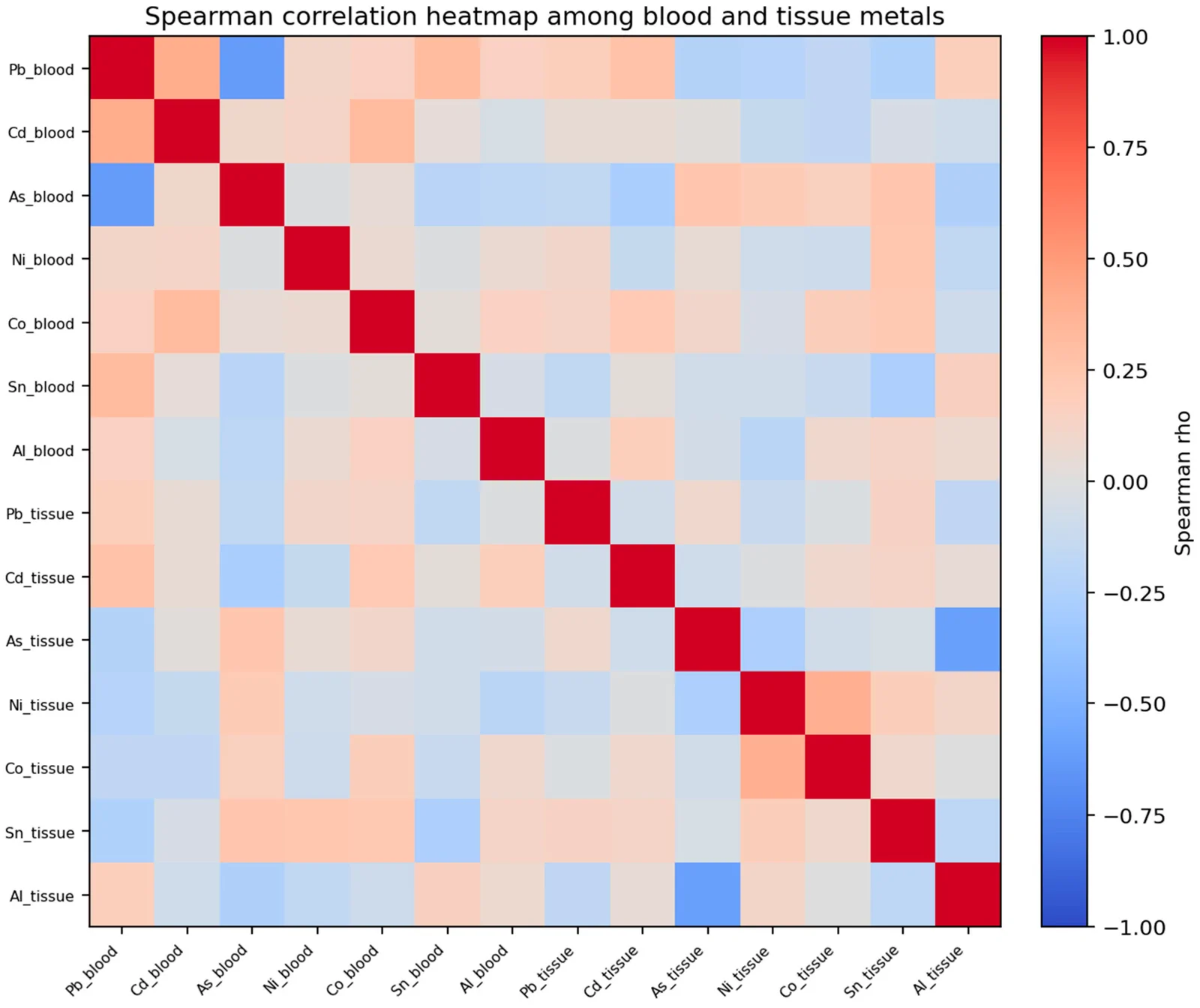
Spearman correlation heatmap across blood and gastric tissue metals.

**Figure 7:**
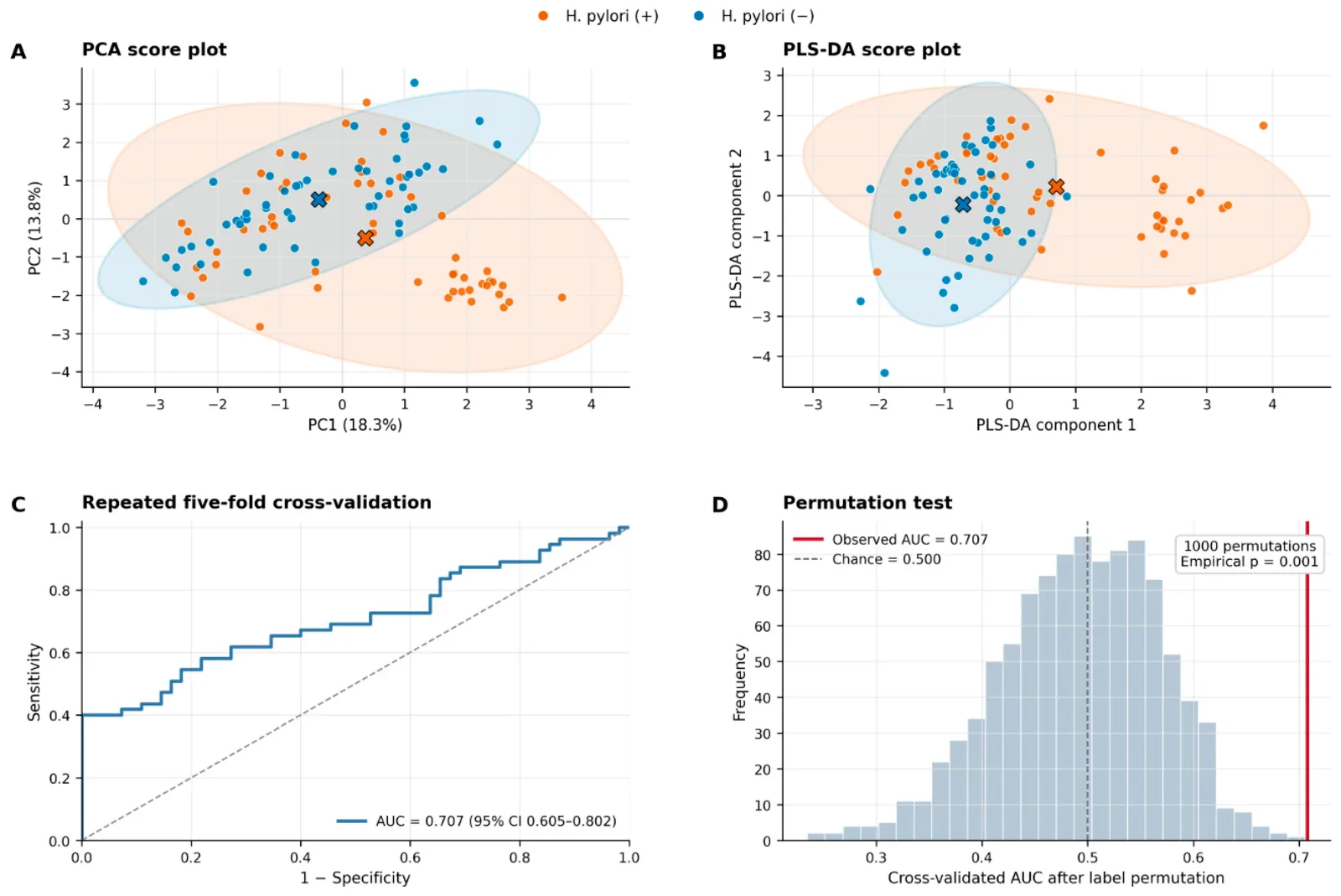
Multivariate separation and internal validation of multi-element profiles according to *H. pylori* infection status. (**A**) Unsupervised principal component analysis (PCA) score plot; PC1 and PC2 explained 18.3% and 13.8% of the total variance, respectively (32.1% cumulatively). (**B**) Supervised partial least squares-discriminant analysis (PLS-DA) score plot. (**C**) Receiver operating characteristic curve derived from repeated five-fold cross-validation, yielding an AUC of 0.707 (95% CI, 0.605–0.802); the dashed diagonal line indicates chance-level discrimination (AUC = 0.5). (**D**) Distribution of cross-validated AUC values generated from 1000 label permutations; the observed AUC of 0.707 exceeded the null distribution (empirical *p* = 0.001); the red solid line indicates the observed AUC, and the dashed line indicates the chance level (AUC = 0.500). Each point represents one participant, shaded ellipses indicate 95% confidence regions, and X symbols denote group centroids. Orange and blue indicate *H. pylori*-positive and *H. pylori*-negative participants, respectively.

Spearman analysis evaluated matched blood-tissue concentrations as a separate family of seven tests (Figure 6). Blood and tissue arsenic were weakly positively correlated (ρ = 0.237; *p* = 0.0128; q = 0.0447), whereas blood and tissue tin were weakly inversely correlated (ρ = −0.258; *p* = 0.0065; q = 0.0447). No other matched-element correlation survived FDR correction. PCA showed a modest centroid shift with substantial overlap; PC1 and PC2 explained 18.3% and 13.8% of variance, respectively. PLS-DA yielded a repeated five-fold cross-validated AUC of 0.707 (95% CI 0.605–0.802). The observed AUC exceeded the null distribution in 1000 label permutations (empirical *p* = 0.001), indicating a non-random within-cohort signal but not clinical utility or external generalizability (Figure 7).

## 4. Discussion

The principal finding was a compartment-specific association between *H. pylori* status and two gastric tissue elements after FDR correction: tissue aluminum was higher and tissue arsenic was lower in the positive group. Blood aluminum, tissue cobalt, and blood/tissue nickel showed only nominal or borderline differences, and the nominal blood aluminum and blood nickel associations were further attenuated after age and sex adjustment. The infected group was also significantly older, so age and sex were included in prespecified core models. The routine biochemical measurements (calcium and TSH) had *p* = 0.050 and are reported as borderline significant.

The stronger group differences in tissue than in blood-derived matrices support the value of paired local and systemic measurement, but they do not establish that gastric tissue is a superior diagnostic matrix. A total tissue concentration can reflect several processes including recent oral contact, epithelial uptake, systemic delivery, altered mucosal permeability, or local retention, and ICP-MS cannot distinguish among these sources. Accordingly, the findings are framed as associations with infection status rather than evidence that environmental exposure caused infection or that infection caused metal accumulation.

The inverse arsenic result was verified directly against the participant-level dataset. *H. pylori*-positive participants had lower tissue arsenic, consistent with the age- and sex-adjusted OR below 1. Because only total arsenic was measured, this unexpected association cannot be assigned to inorganic, methylated, or organic species and should not be interpreted as protective. The aluminum distribution was strongly right-skewed, and higher tissue aluminum likewise does not demonstrate chronic accumulation or gastric toxicity. For both elements, unmeasured oral exposure, tissue handling, local physiology, and residual geographic or socioeconomic confounding remain plausible explanations.

Nickel requires separate host–pathogen consideration. *H. pylori* urease uses nickel to generate ammonia and bicarbonate for acid acclimation, while [NiFe]-hydrogenase enables use of molecular hydrogen as an energy source. Nickel uptake through NixA and NiuBDE, delivery by urease/hydrogenase accessory proteins, storage by histidine-rich proteins, and NikR-mediated regulation balance enzyme activation against metal toxicity; experimental disruption of nickel transport or hydrogenase reduces colonization in animal models [22,23,24,26]. At the host interface, calprotectin can sequester nickel and restrict access to microbial metalloenzymes [25]. These mechanisms are relevant context, but the observed tissue nickel difference (*p* = 0.065; q = 0.170) and blood nickel difference (*p* = 0.073; q = 0.170) were not significant after multiplicity correction. Total-tissue ICP-MS cannot distinguish bacterial, host-bound, extracellular, or bioavailable nickel fractions, so no mechanistic conclusion is drawn from these borderline findings.

The regression, ROC, PCA, and PLS-DA analyses were exploratory. In the joint logistic model, tissue aluminum remained associated with positivity, whereas tissue arsenic was attenuated after simultaneous adjustment. The apparent AUC of 0.774 and cross-validated PLS-DA AUC of 0.707 indicate modest within-cohort discrimination. The permutation result supports a multielement signal beyond random class labels, but it does not establish diagnostic accuracy, causality, or transportability to another population.

Study strengths include histopathological confirmation of active *H. pylori* infection, paired blood-derived and fresh gastric tissue measurements, explicit separation of histology and analytical specimens, multi-element ICP-MS, and transparent multiplicity correction. These features reduce disease misclassification and permit direct comparison of compartments within participants. Nevertheless, the study was designed to identify associations and generate hypotheses, not to establish exposure sources or mechanisms.

Several limitations should be considered. The cross-sectional, single-center design precludes temporal and causal inference, and the modest sample limited model stability (55 positive events; 9.2 events per variable in the exploratory model). Detailed birthplace, childhood residence, rural/urban status, drinking-water source, diet, occupation, and socioeconomic data were unavailable, leaving substantial potential for residual confounding. Recent medication use, including proton-pump inhibitors, antibiotics, bismuth, and prior eradication therapy, was not comprehensively characterized. Although fasting and rinsing reduced luminal contamination, recent oral contact or surface-adherent material cannot be excluded. Total concentrations do not provide arsenic speciation or nickel bioavailability, and the markedly skewed tissue aluminum distribution increases sensitivity to influential observations. Histopathological severity, bacterial density, oxidative-stress biomarkers, cytokines, epithelial injury, and toxicological thresholds were not assessed. Finally, internal cross-validation does not replace independent external validation.

## 5. Conclusions

In conclusion, histopathologically confirmed *H. pylori* infection was associated with higher gastric tissue aluminum and lower gastric tissue arsenic after FDR correction, while the nickel findings were borderline and non-significant after correction. These concentration differences do not demonstrate environmental exposure, chronic accumulation, organ toxicity, or causality. Independent multicenter studies should combine standardized fresh-biopsy collection and weight normalization with arsenic speciation, external exposure data, quantitative histopathology, bacterial-density measures, and molecular effect biomarkers.

## Data Availability

The original contributions presented in this study are included in the article. Further inquiries can be directed to the corresponding author.
